# Nanoparticles Synthesis in Wet-Operating Stirred Media: Investigation on the Grinding Efficiency

**DOI:** 10.3390/ma13194281

**Published:** 2020-09-25

**Authors:** Marco Trofa, Gaetano D’Avino, Bruno Fabiano, Marco Vocciante

**Affiliations:** 1 Dipartimento di Ingegneria Chimica, dei Materiali e della Produzione Industriale, Università di Napoli Federico II, Piazza Giorgio Ascarelli 80, 80125 Napoli, Italy; marco.trofa@unina.it (M.T.); gadavino@unina.it (G.D.); 2 Department of Chemical, Civil and Environmental Engineering, University of Genova, Via Opera Pia 15, 16145 Genova, Italy; brown@unige.it; 3 Department of Chemistry and Industrial Chemistry, University of Genova, Via Dodecaneso 31, 16146 Genova, Italy

**Keywords:** numerical simulations, computational fluid dynamics, nanoparticle synthesis, bead milling, top-down method, magnetic stirring, eco-friendly process

## Abstract

The use of nanomaterials, thanks to their peculiar properties and versatility, is becoming central in an increasing number of scientific and engineering applications. At the same time, the growing concern towards environmental issues drives the seeking of alternative strategies for a safer and more sustainable production of nanoparticles. Here we focus on a low-energy, magnetically-driven wet milling technique for the synthesis of metal nanoparticles starting from a bulky solid. The proposed approach is simple, economical, sustainable, and provides numerous advantages, including the minimization of the nanoparticles air dispersion and a greater control over the final product. This process is investigated by experiments and discrete element method simulations to reproduce the movement of the grinding beads and study the collision dynamics. The effect of several parameters is analyzed, including the stirring bar velocity, its inclination, and the grinding bead size, to quantify the actual frequency, energy, and angle of collisions. Experiments reveal a non-monotonous effect of the stirring velocity on the abrasion efficiency, whereas numerical simulations highlight the prevalent tangential nature of collisions, which is only weakly affected by the stirring velocity. On the other hand, the stirring velocity affects the collision frequency and relative kinetic energy, suggesting the existence of an optimal parameters combination. Although a small variation of the stirring bar length does not significantly affect the collision dynamics, the use of grinding beads of different dimensions offers several tuning opportunities.

## 1. Introduction

The synthesis of nanoparticles (NPs) and nanostructured materials has received progressively growing attention in recent years thanks to their versatility in a wide variety of technical applications, ranging from the medical [1] to industrial [2] and environmental [3] field. Many manufacturing processes can be adopted depending on the compound to be synthesized, which in general can be grouped into two opposite strategies, i.e., bottom-up and top-down methods. So far, the first approach has attracted much more attention as the NPs size is easier to monitor during growth than during disaggregation [4]. Very often, chemical methods of this type are a first-rank choice in the synthesis of nanomaterials for environmental remediation [5], biomedicine [6], electronic engineering [7], and so on. Indeed, zerovalent NPs of noble and weakly-electropositive elements have been successfully produced by redox processes [8,9]. The main drawback related to bottom-up methods is the use of various chemicals (as reagents, complexing agents, and surfactants) having toxic effects on human health and the environment. For this reason, research is increasingly pushing towards the development of new approaches that are cheaper, intrinsically safer, and more environmentally sustainable. Such a goal can be achieved by adopting new procedures [10] that generally refer to ‘green nanotechnology’ [11], in which reagents derived from plant extracts, pigments, yeast, and enzymes are employed [12,13,14].

Recently, top-down methods have also been developed [15], whereby NPs are formed starting from elements or compounds in macroscopic sizes undergoing physical treatments without any chemical reaction. Several disaggregation techniques have been proposed [16], mainly depending on the presence/absence of milling media like spheres or beads in a vessel containing the phase subject to comminution [17]. In some cases, stabilizing agents are also employed to hinder the re-aggregation of produced nanoparticles [18]. One of the drawbacks of such methods is the lack of control on the NPs size (broad distribution), thus requiring the adoption of specific strategies.

To obtain more homogeneous NPs (with reduced size distribution), we consider a top-down method based on the mechanical refinement of silver (Ag) spheres in wet-operating stirred media [15]: Ag spheres, dispersed in an aqueous solvent with capping agents, are abraded by impact with yttria-stabilized zirconia beads. Owing to the solvent presence, the disaggregation process directly releases zerovalent Ag nanoparticles as a dispersed phase, thus minimizing NPs air dispersion and leading to greater control over the final product. Indeed, such simple innovation leads to a more eco-friendly and economically sustainable NPs synthesis.

Notice that studies proposing a one-pot bead milling process where NPs are produced directly starting from bulky solid-like metal spheres of a millimetric dimension are quite uncommon in literature. As a matter of fact, low-energy bead-milling media-assisted apparati are generally used to decrease the diameter of particles already belonging to a micron or even submicron range (i.e., disaggregating NP agglomerates in two-pots processes [19]), following the commonly accepted rule of thumb that the minimum achievable final diameter of produced NPs is 1/1000 of the primary particles. On the other hand, the milling technique here investigated has been shown to be able to reach a comminution ratio of $1/10^{5}$, exploiting a peculiar setup configuration [4].

In this contribution, the aforementioned process is investigated by experiments and numerical simulations. Due to the high particle volume fraction and confinement of the setup, a Discrete Element Method (DEM) is employed to track the movement of grinding beads [20,21]. The effect of several parameters has been studied including the stirring velocity, the inclination of the magnetic anchor, and the size distribution of the beads. The results are presented in terms of frequency, energy, and angle of collision.

## 2. Materials and Methods

### 2.1. Experimental Set-Up

**Figure 1:**
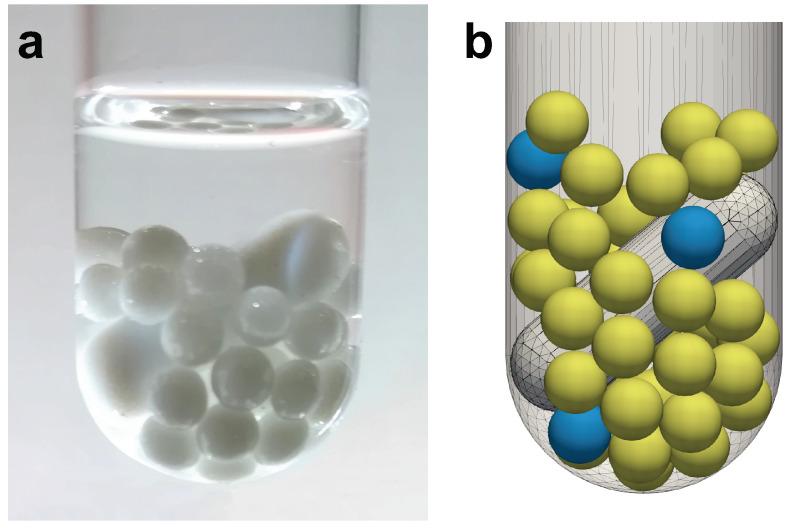
(**a**) Detail of the experimental setup. (**b**) Snapshot of the bottom portion of the simulation domain, with stirring bar and the beads (yellow for ZrO${}_{2}$ and light blue for Ag), partially lifted by the bar motion.

The reference experimental setup, shown in Figure 1a, consists of: (i) A cylindrical glass container (inner diameter of 13 mm) with a hemispherical base; (ii) a cylindrical polytetrafluoroethylene (PTFE) coated magnetic bar (diameter of 4.6 mm and length of 15 mm) with hemispherical ends, assuming a slanted position inside the container at 40 degrees from the horizontal; (iii) 4 silver beads (a diameter of 3 mm, Ag 99.9%, American Elements, Los Angeles, USA) as metal precursor undergoing disaggregation; and (iv) 40 beads of yttria-stabilized zirconia (a diameter of 3 mm, ZrO${}_{2}$ 95%, Y${}_{2}$O${}_{3}$ 5%, MSE Supplies, Tucson, AZ, USA) as grinding media (chosen for its high surface hardness and known anti-scratch properties). Solvent is added so as to obtain a meniscus 5 mm above the particles packed bed, ensuring a complete immersion of the beads as well as when the bed slightly expands due to stirring. On the other hand, a too high liquid level may disperse the produced NPs and make their recovery more difficult. Previous investigations have shown that this configuration avoids the stirring bar to get stuck, whereas such phenomenon can occur by increasing the number of spheres or their dimension. The detailed description of the experimental apparatus mentioned above can be found in Reference [22].

Experiments have been conducted to assess the optimal stirring velocity for the production of NPs: Several sets of fresh beads have been used to run experiments of increasing duration (from 1 to 4 h) at 3 different stirring velocities (300, 600, and 900 rpm). Higher rotational speeds cause the stirring bar to rise up inside the bed with consequent loss of efficiency. To verify the overall abrasion effectiveness of the system, after each experiment, the beads were washed with alcohol to facilitate evaporation, dried at room temperature, and finally weighed to evaluate the mass of silver lost during the process. A SEM analysis on the produced NPs was also conducted on some of the samples.

### 2.2. Numerical Set-Up

The dynamics of the grinding system, governed by beads collisions, has been reproduced by DEM numerical simulations. The computational domain is schematically reported in Figure 1b. The yellow and light blue beads denote the ZrO${}_{2}$ and Ag particles, respectively. The container and stirring bar surfaces are discretized through a triangular mesh, used to determine the contacts with the particles. Positions and velocities are defined with respect to a Cartesian reference frame, having the origin in the center of the container hemispherical base and the *z*-axis along the axis of the cylindrical section of the container. The system movement is determined by the rotation around the container axis of the bar mesh.

The DEM method is a Lagrangian model allowing one to track the particles by explicitly solving their trajectories [23]. The *i*-th particle position $x_{i}$ and angular position $\theta_{i}$ are updated by integrating the following kinematic equations:

(1) $$\frac{dx_{i}}{dt}=u_{i}\;\;\frac{d\theta_{i}}{dt}=\omega_{i}$$

with $u_{i}$ and $\omega_{i}$ the particle translational and angular velocities. We adopt the usual initialization procedure to let the particle settle from a bigger volume to obtain a random distribution in high volume fraction systems [20,21], with initial values $u_{i,0}=\theta_{i,0}=\omega_{i,0}=0$. The particle translational and angular velocities are computed from the force and torque balances:

(2) $$m_{i}\frac{du_{i}}{dt}=\sum_{j}F_{c}+m_{i}g\;\;I_{i}\frac{d\omega_{i}}{dt}=\sum_{j}T_{c}$$

where $m_{i}$ and $I_{i}$ are the particle mass and moment of inertia, g is the gravity force pointing towards the negative *z*-direction, and $F_{c}$ and $T_{c}$ are the force and torque acting on a given particle due to the *j*-th contacts with other particles or the walls.

The impact between two rigid spherical particles is collinear, i.e., there is a single contact point located on the segment joining the particle centers, and such a point identifies a common tangent plane of unit normal vector n (pointing from *j* to *i*). From the relative velocity between the contacting particle surfaces $v_{c}=v_{i}-v_{j}$, where $v_{i}=u_{i}+\omega_{i}\times r_{i}$ is the velocity of the particle surface and $r_{i}$ is the position vector from the particle center to the contact point, we can also define a unit tangential vector t and a relative tangential velocity $v_{c,t}=\left(I-\mathrm{nn}\right)\;\cdot \;\left(u_{i}-u_{j}\right)-\left(r_{i}\omega_{i}+r_{j}\omega_{j}\right)\times n$ with I the unity tensor, and $r_{i}$ and $r_{j}$ the radii of the particles.

The contact force and torque are calculated through the Hertz–Mindlin model [24]:

(3) $$\begin{array}{c} F_{c}=F_{c,n}n+F_{c,t}t=\left(k_{n}\delta_{n}+\gamma_{n}\frac{d\delta_{n}}{dt}\right)n-\left(k_{t}\delta_{t}+\gamma_{t}\frac{d\delta_{t}}{dt}\right)t \end{array}$$

(4) $$\begin{array}{c} T_{c}=-r_{i}F_{c,t}\;n\times t. \end{array}$$

The tangential component of the contact force $F_{c,t}$ is constrained by the well-known Coulomb law of friction $\left|F_{c,t}\right|\leq \mu_{F}\left|F_{c,n}\right|$, in which $\mu_{F}$ is the sliding friction coefficient. The other symbols are defined in Table 1 where ϵ is the coefficient of restitution.

The properties of the materials considered are listed in Table 2 and have been taken from the literature (see Reference [25] for zirconia and glass, Reference [26] for silver, and Reference [27] for PTFE).

A time step of 10^−7^s, much smaller than the actual collision time [20,21], ensures simulation stability and full resolution of the particle contacts. Due to the aforementioned initialization procedure, the simulations are run for 0.5 s to let the system settle and reach a pseudo steady-state condition. In the following 0.5 s, the new impacts between the particles and detachments are monitored at every time step, allowing the determination of collision frequency and duration.

In every collision, the energy available for abrasion is the kinetic energy $ke_{r}$ connected to the relative velocity between the contacting particle surfaces at the beginning of the contact (which has normal and tangential components $v_{c,n}$ and $v_{c,t}$):

(5) ker=12vcT·M·vc=12vc,tvc,n·1mi+1mj−12/7001·vc,tvc,n

where M is the inertia matrix (symmetric positive definite) of the two spherical particles system [28]. In Equation (5), the coefficient 2/7 is related to the gyration radius of spheres. Finally, an impact angle can be defined as:

(6) $$\theta_{\mathrm{impact}}=\arctan\left(\frac{v_{c,n}}{v_{c,t}}\right)$$

which is the angle between the initial relative contact velocity and the common tangent plane, i.e., $\theta_{\mathrm{impact}}=90{}^{\circ}$ for a direct impact, parallel to the normal direction.

To get rid of the variability introduced by the initialization procedure, for each set of operative parameters, we ran several simulations with different initial random particle distributions and took the average results. The number of repeated simulations is selected such that the ensemble of the results is characterized by a standard deviation lower than a tolerance of 5%. The simulations have been implemented through the open-source software LIGGGHTS 3.8.0 (described in detail in Reference [29]).

## 3. Results

### 3.1. Experimental Etching Efficiency

**Figure 2:**
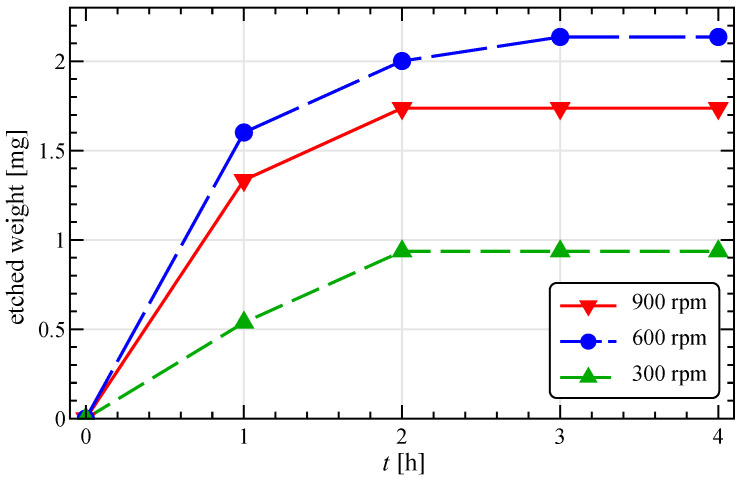
Experimental time evolution of the total etched material from the Ag beads at different stirring velocities. The initial total mass of the four Ag beads is about 593 mg.

Figure 2 represent the time evolution of the mass of etched material from the four Ag particles, obtained by weighting several sets of fresh beads in experiments of increasing duration (from 1 to 4 h) at three different stirring velocities (300, 600, and 900 rpm).

The data show a progressive loss of effectiveness in the abrasion process, as the quantity of material removed at a given rotation speed gradually reaches a plateau value, i.e., the abrasion velocity decreases towards zero. This phenomenon is probably linked to changes in the surface hardness of the silver beads, which, interestingly, seems to be a temporary condition. Indeed, preliminary experiments have demonstrated that ‘exhausted’ beads, let stand for about 72 h, partially recover their abrasion capability. Further and more accurate tests are underway, although the investigation of the changes in the superficial properties of the primary particles lies outside the purpose of our simulation work, which focuses on short times (at the very start of the process) and then only accounts for constant material properties.

From Figure 2 it is also evident that the stirring velocity has a non-monotonous effect on the abrasion efficiency, as the maximum quantity of etched mass is obtained at the intermediate velocity of 600 rpm. Assuming the NPs produced are proportional to the etched mass and present a similar size distribution in all the investigated conditions, the experimental data reported suggest 600 rpm as the optimal operative condition between the values investigated.

### 3.2. Numerical Characterization of Collisions

**Figure 3:**
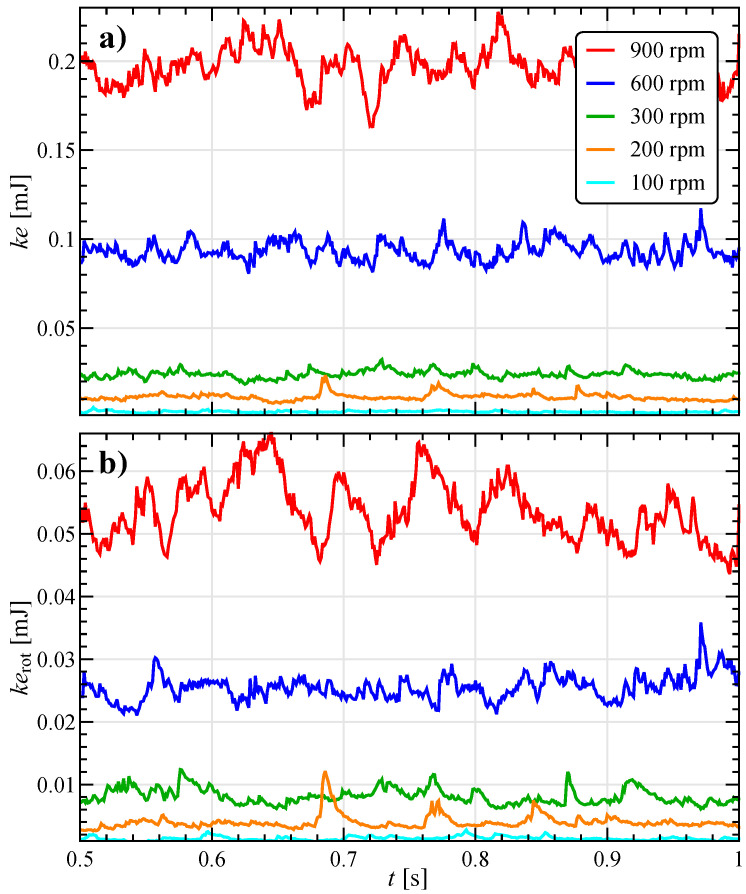
Total (**a**) translational and (**b**) rotational kinetic energy for the reference system at different stirring velocities in a time window of 0.5 s after the initialization transient.

The initialization procedure discussed above determines a fast initial transient followed by a pseudo steady-state regime [20,21]. The attainment of such a regime is testified by the trends of the translational and rotational kinetic energy of all the particles in the system ($ke=0.5\sum m_{i}u_{i}^{2}$ and $ke_{\mathrm{rot}}=0.5\sum I_{i}\omega_{i}^{2}$) reported in Figure 3 at different stirring velocities Ω. The data are an average over several simulations with different initial random particle distributions. Both quantities present some fluctuations with mean and variance nearly constant in time, which are proportional to the stirring velocity. As a time scale reference, a complete bar rotation around the vial axis takes 0.1 s at 600 rpm, corresponding to five revolutions in the time window shown. Notice that, in addition to the stirring velocities considered in the experiments, 100 and 200 rpm are also investigated numerically, to better clarify the effect of Ω.

**Figure 4:**
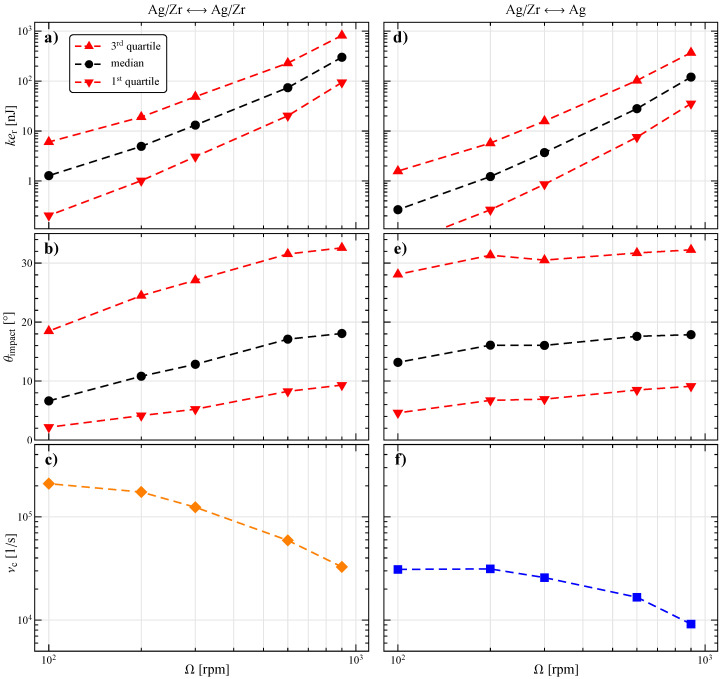
(**a**,**d**) Relative kinetic energy, (**b**,**e**) impact angle, and (**c**,**f**) collision frequency as a function of the stirring velocity for the impacts between all the particles (left column) and those involving at least an Ag particle (right column). In the first and second row the median, first, and third quartiles of the distribution are shown.

Considering all the collisions in the system, the distribution of the relative kinetic energy $ke_{r}$ and impact angle $\theta_{\mathrm{impact}}$ are characterized by the quartiles shown in Figure 4, where the average collision frequency $\nu_{c}$ is also reported. The left column (panels a, b, and c) refers to the impacts between all the particles, whereas the right column (panels d, e, and f) is for those involving at least an Ag particle.

As expected, an increase in the relative kinetic energy is observed by increasing the stirring velocity. In particular, as evident by the graph in log-log scale, the data can be described by a power-law more than quadratic (about $ke_{r}\propto \Omega^{2.5}$). This implies that the relative velocity between the particle surfaces in not directly proportional to Ω. The log scale is also helpful to distinguish the energy distribution, which spans different orders of magnitude. If we consider the collisions involving at least an Ag particle (panel d) we notice a similar trend, but with a significant reduction in $ke_{r}$ of a factor around three. Hence, although Ag particles have a higher mass, which should determine an increase in the kinetic energy, its effect is compensated and overcome by a reduction in the relative velocity.

Concerning the impact angle, firstly we notice that, in the range of stirring velocities considered, its distribution is shifted towards lower values, indicating mainly tangential collisions. Indeed, 75% of the data (i.e., those before the 3rd quartiles) is below about 30°, with a median always below 20°. This result is positive for the production of NPs as it has been shown that ductile materials (like Ag) present a maximum abrasion efficiency at low impact angles [30,31,32]. The impact angle $\theta_{\mathrm{impact}}$ increases with Ω, probably because the particle bed expands, increasing the relative distance between particles, giving rise to more direct collisions. The trend of $\theta_{\mathrm{impact}}$ involving at least an Ag particle (panel e) is more constant. Notice, however, that the two curves in panels b and e can be nearly superimposed in the range of experimental interest, i.e., with stirring velocities between 300 and 900 rpm.

Since the variation of the impact angle is small, the behavior of the kinetic energy would suggest to maximize the stirring velocity to enhance abrasion. However, this would determine a reduction of the impact frequency (panels c and f), also connected to the expansion of the particle bed, ultimately decreasing the number of NPs produced. This consideration helps to explain the experimental results reported in Figure 2, where an Ω of 900 rpm determines a lower etched weight as compared to 600 rpm.

It is worth noticing that, just like for the impact angle, there is an optimal operative range also for the relative kinetic energy. Too low $ke_{r}$ are not able to induce abrasion (removal of small surface portions), whereas too high values may cause plastic deformations or the production of big fragments that cannot be efficiently comminuted to a nanoscopic size.

**Figure 5:**
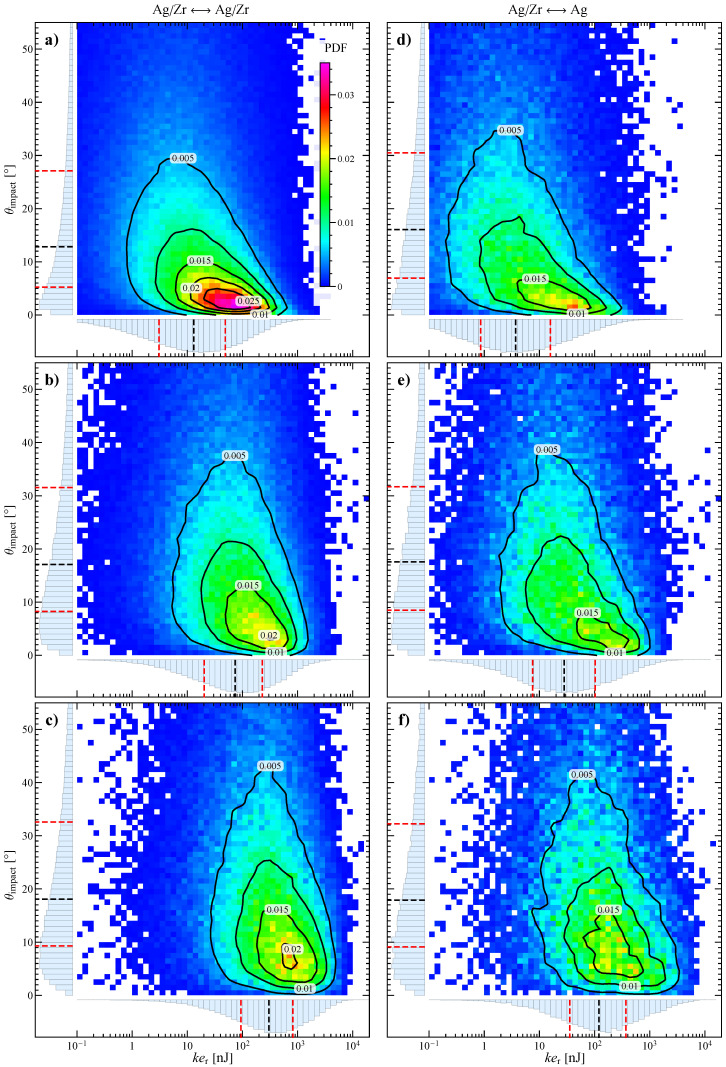
Map and contours of the joint probability density function for impact angle and relative kinetic energy at (**a**,**d**) 300 rpm, (**b**,**e**) 600 rpm, and (**c**,**f**) 900 rpm for the impacts between all the particles (left column) and those involving at least an Ag particle (right column). On the edges of every map the marginal probability density functions and the corresponding quartiles are also shown (the dashed red lines representing the first and third quartiles and the dashed black line the median).

To better characterize the collisions and identify the optimal operative conditions, in Figure 5 we report the joint probability density function (PDF) (with color map and contours) for the impact angle and logarithm of relative kinetic energy. On the edges of every map the marginal probability density functions and the corresponding quartiles (the same of Figure 4a,b) are also shown. The binning is every 1° for $\theta_{\mathrm{impact}}$ and every 0.1 for $\log(ke_{r})$, hence the probability of finding a collision with parameters in a specific 2D bin is the value of the bin multiplied by its area, i.e., 0.1.

From Figure 5 it is possible to notice that the joint distributions are all unimodal and the most probable collisions have a kinetic energy higher and impact angle lower than the corresponding medians (this is particularly evident in panel a). In addition, there is a negative correlation between $\theta_{\mathrm{impact}}$ and $ke_{r}$, i.e., the higher the angle, the lower the energy. Furthermore, the marginal distribution of $\theta_{\mathrm{impact}}$ has a significant positive skewness (with a long tail at high angles), hence, although most impacts are mainly tangential, some direct impacts also take place. As seen in Figure 4, at increasing stirring velocity (see panel b and c of Figure 5) the relative kinetic energy substantially increases (the distribution shift to the right), whereas the impact angle is more spread and uniform, ultimately reducing the negative correlation and producing a more symmetric distribution. Notice that, the maps and contours corresponding to Ag/Zr ↔ Ag collisions (right column in Figure 5), although more scattered due to the lower number of data, approximately reproduce the same trend of the whole collisions (left column), simply shifted at a lower $ke_{r}$.

By recalling the experimental results of Figure 2 at varying Ω, it is now evident the presence of a region identifying the best conditions in terms of the impact angle and kinetic energy discussed above. However, in a global optimization procedure, it is also necessary to take into account the negative effect of the stirring velocity on the impact frequency.

### 3.3. Influence of the Stirring Bar Inclination

**Figure 6:**
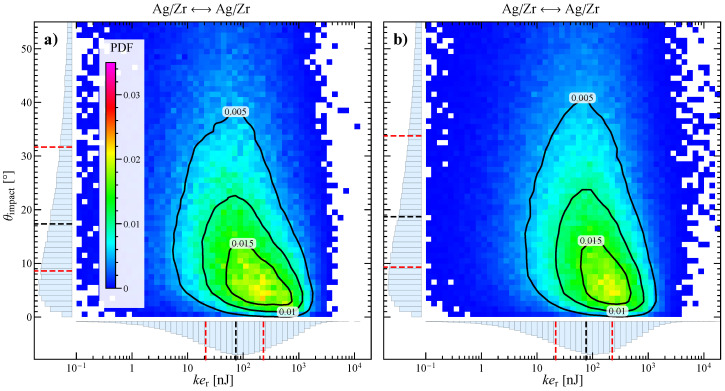
Map and contours of the joint probability density function for impact angle and relative kinetic energy for the impacts between all the particles at 600 rpm and with stirring bar angle of (**a**) 30° and (**b**) 50°. On the edges of every map the marginal probability density functions and the corresponding quartiles are also shown (the dashed red lines representing the first and third quartiles and the dashed black line the median).

In Figure 6, the effect of the inclination of the stirring bar on the relative kinetic energy and impact angle is reported. A small variation of the stirring bar inclination in the particle bed (±10°), corresponding to bars of different length, does not significantly affect the collision dynamics. Indeed, the two panels of figure, showing the joint probability density functions for systems at 600 rpm with a bar angle of 30° and 50°, are both very similar to the reference case. The same behavior occurs for the other two stirring velocities investigated (not reported).

### 3.4. Influence of the Bead Size

**Figure 7:**
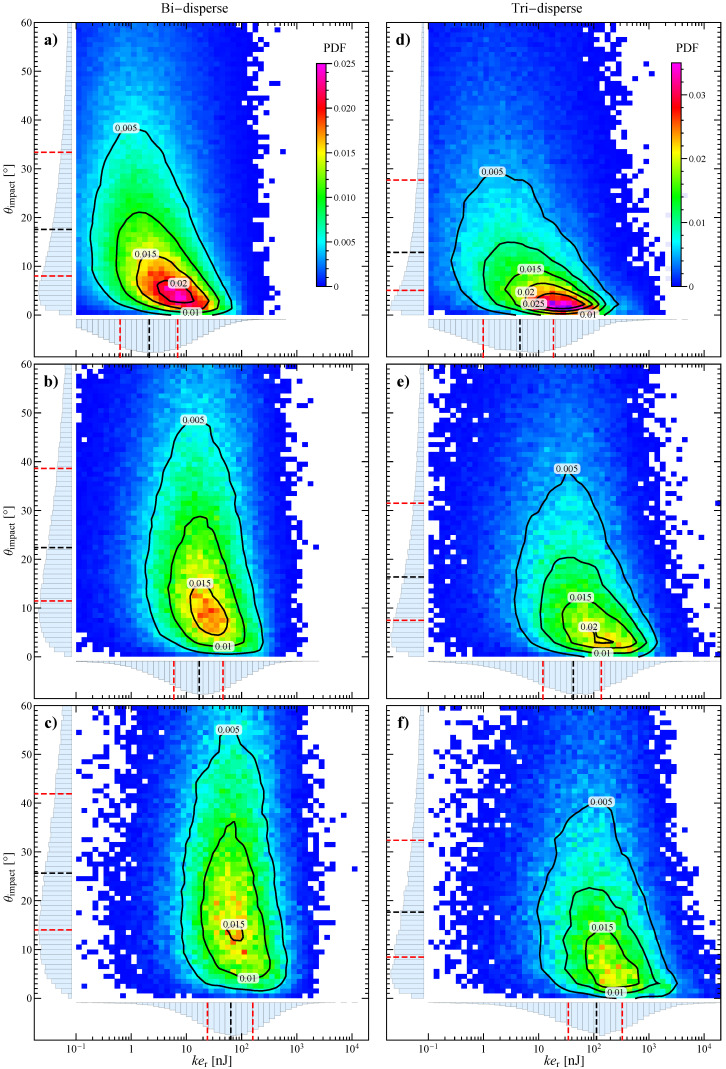
Map and contours of the joint probability density function for impact angle and relative kinetic energy at (**a**,**d**) 300 rpm, (**b**,**e**) 600 rpm, and (**c**,**f**) 900 rpm for the impacts between all the particles in the bi-disperse (left column) and tri-disperse system (right column). On the edges of every map the marginal probability density functions and the corresponding quartiles are also shown (the dashed red lines representing the first and third quartiles and the dashed black line the median).

A crucial aspect in bead milling processes is the balancing between the size of the grinding beads and that of the particles to be treated, as a relatively large inter-bead space at a close-packing configuration may determine a dramatic reduction of milling efficiency [22]. To this aim we performed further simulations by varying the dimension and number of the grinding beads, to reproduce different bed configurations. In particular, we investigated a bi-disperse system, keeping fixed the Ag particles (four particles of 3 mm diameter) and the total solid volume fraction, with 135 ZrO${}_{2}$ particles of 2 mm. Furthermore, we also considered a tri-disperse system with the same Ag particles, 55 ZrO${}_{2}$ particles of 2 mm and 10 of 4 mm. The corresponding results in terms of joint probability density function are reported in the two columns of Figure 7.

**Figure 8:**
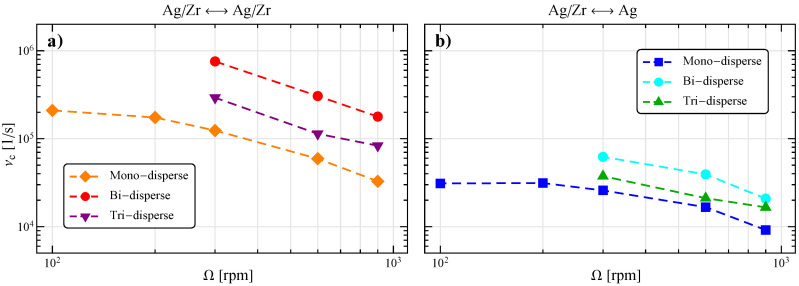
Collision frequency as a function of the stirring velocity for the impacts between all the particles (**a**) and those involving at least an Ag particle (**b**).

At every Ω, the bi-disperse system shows a reduction of the relative kinetic energy and a spreading of the impact angle towards higher values as compared to the reference case in Figure 5. The first can be partially explained by the lower mass of the grinding beads (by a factor ${\left(2/3\right)}^{3}$) that, due to the higher number of beads, now dominates even further the collisions. The second could be connected to a reduction of the particle angular velocity, but the identification of the actual cause is hindered by the complex intertwining between particle velocities (translational and rotational) and positions in the determination of the components of the relative surface velocity for every collision. Obviously, due to the increased number of grinding particles in the system, the collision frequency, both total and with at least an Ag particle, is enhanced (see Figure 8).

The tri-disperse system seems to recover the behavior of the reference one, probably because the 55 smaller and 10 bigger particles compensate their effect. However, the collision frequency is higher as compared to the reference case due to the higher particle number.

At increasing stirring velocities, the particle collisions become more energetic, but also less tangential and frequent, therefore an optimal condition needs to be determined. By varying the size and number of grinding beads, it is possible to increase the collision frequency at the expense of the impact energy and angle. Such a loss can be compensated in a system composed by two or more particle dimensions. However, the optimization becomes more and more complex due to the increased number of parameters, not least the dimensional ratio with the container as the particle bed is highly confined.

## 4. Conclusions

Experiments and numerical simulations on a wet-operating ball mill have been performed. The effect of several parameters has been studied including the stirring velocity, the stirring bar angle, and the grinding bead size, with an in-depth impact analysis to enable a quantification of the actual frequency, energy, and angle of collisions. The average values from several simulations with different initial random particle distributions have been considered to ensure statistical invariance.

Experiments have shown that the stirring velocity has a non-monotonous effect on the abrasion efficiency, highlighting the importance of this parameter and suggesting further investigation. DEM simulations have confirmed that the system dynamics is stable in the whole range of stirring velocities considered. The impact angle is generally below 30° indicating mainly tangential collisions, which have been proved to be more effective in the abrasion of metal particles. By increasing the stirring velocity, the relative kinetic energy increases, following a power-law scaling more than quadratic. The impact angle also grows but the variation is small, and the collision frequency decreases due to the expansion of the particle bed.

A small variation of the stirring bar length, and consequently its inclination in the particle bed (±10°), does not significantly affect the collision dynamics, thus enhancing reproducibility of experiments. On the other hand, the use of grinding beads of different dimensions offers several tuning opportunities, as playing with the size and number of grinding beads allows to modify collision frequency, impact energy, and angle, at a given stirring velocity and/or solid volume fraction. However, due to the increased number of parameters, finding the optimal set of parameters becomes experimentally costly (in terms of time and materials). In this context, numerical simulations can represent a useful resource to understand the effect of the various parameters and guide optimization towards a development of new, more efficient, inherently safe, and eco-friendly nanoparticle production process.

## Figures and Tables

**Table 1:** Equations for the Discrete Element Method (DEM) model. Notice that for particle-wall interactions the equivalent radius and mass are those of the particle.

Parameter	Equation
Equivalent radius	$r_{r}={\left(\frac{1}{r_{i}}+\frac{1}{r_{j}}\right)}^{-1}$
Equivalent mass	$m_{r}={\left(\frac{1}{m_{i}}+\frac{1}{m_{j}}\right)}^{-1}$
Equivalent Young modulus	$E_{r}={\left(\frac{1-\nu_{i}^{2}}{E_{i}}+\frac{1-\nu_{j}^{2}}{E_{j}}\right)}^{-1}$
Equivalent shear modulus	$G_{r}={\left(\frac{2\left(1+\nu_{i}\right)\left(2-\nu_{i}\right)}{E_{i}}+\frac{2\left(1+\nu_{j}\right)\left(2-\nu_{j}\right)}{E_{j}}\right)}^{-1}$
Normal overlap	$\delta_{n}=r_{i}+r_{j}-∥x_{i}-x_{j}∥$
Tangential overlap	$\delta_{t}=\int_{t_{0}}^{t}v_{c,t}\;\cdot \;t\;d\tau$
Normal stiffness	$k_{n}=\frac{4}{3}E_{r}\sqrt{r_{r}\delta_{n}}$
Tangential stiffness	$k_{t}=8G_{r}\sqrt{r_{r}\delta_{n}}$
Normal damping coefficient	$\gamma_{n}=-2\frac{\ln\epsilon }{\sqrt{\ln\epsilon^{2}+\pi^{2}}}\sqrt{\frac{5}{3}m_{r}E_{r}\sqrt{r_{r}\delta_{n}}}$
Tangential damping coefficient	$\gamma_{t}=-4\frac{\ln\epsilon }{\sqrt{\ln\epsilon^{2}+\pi^{2}}}\sqrt{\frac{5}{3}m_{r}G_{r}\sqrt{r_{r}\delta_{n}}}$

**Table 2:** Model parameters and material properties (adapted from [21]).

	Zirconia, ZrO${}_{2}$	Silver, Ag	Glass	PTFE
Density [$kg/m^{3}$]	6067	10490	2510	2200
Young’s Modulus [GPa]	210.0	82.0	70.0	0.50
Poisson ratio	0.31	0.36	0.24	0.46
Coefficient of restitution	0.92	0.80	0.99	0.80
Coefficient of friction	0.15	0.55	0.27	0.08
